# Household food insecurity and its associated factors in Ethiopia: a systematic review and meta-analysis

**DOI:** 10.1186/s40795-025-01207-x

**Published:** 2025-11-27

**Authors:** Melesse Belayneh Yayeh, Mastewal Tamiru Assegie, Eshetu Elfios Endrias

**Affiliations:** 1 Department of public health, School of public health, College of Medicine and Health Science, Bahir Dar University, Bahir, Ethiopia; 2 Tibebe Ghion Specialized hospital, College of Medicine and Health Science, Bahir Dar University, Bahir Dar, Ethiopia; 3 Department of Nursing, School of Nursing, College of Medicine and Health sciences, Wolaita Sodo University, Wolaita Sodo, Ethiopia

**Keywords:** Household, Food insecurity, Associated factors, Systematic review, Ethiopia

## Abstract

**Background:**

Household food insecurity continues to be a public health concern, especially in developing countries, including Ethiopia. Despite efforts by the Ethiopian Federal Government to address food insecurity, it has persistently remained one of the top problems. The pooled prevalence of the national is crucial for interventions by clear insight for policy makers, decision makers, and planners of the country by addressing the limited evidence of the household prevalence in Ethiopia. Given the importance of addressing household food insecurity in Ethiopia, there is a growing body of research that examines its prevalence, determinants, and consequences. However, existing evidence is spread across various regions, with variability in the findings. This study aimed to determine the pooled prevalence of household food insecurity and its associated factors in Ethiopia.

**Methods:**

We used PRISMA to identify eligible studies. The inclusion criteria for this study were published in English, publication year, Ethiopian studies, relevant to topics, while editorial letters, complex abstracts and difficult to obtain full texts were excluded from the eligibility criteria. The data was analyzed using STATA version 14 with random-effects model. The forest plot was used to determine the odds ratio for each study of prevalence and associated factors and the general studies with 95% CI presented. The heterogeneity among the studies was checked by I^2^with 95% CI and p-value. Publication bias was checked with funnel plot graphical visualization and statistically using the Egger test.

**Result:**

Examination of 768 studies, ten studies involving 4734 households, and ten out of 768 published articles evaluated satisfied the inclusion criteria and were added to the systematic and meta-analysis. In this systematic review and meta-analysis, the pooled prevalence of household food insecurity of 51.46 % (95% CI: 41.52%, 61.40%). However, subgroup analysis by region was performed in which the highest prevalence of household food insecurity was observed in the Amhara region (61.14% (95% CI 26.00, 68.77) while the lowest prevalence of household food insecurity was observed in the Oromia region (41.52% (95% CI 21.27, 61.78), I2 = 98.9%). Furthermore, this review, pooled factor illustrated that households with larger families were 6.43 times (OR=6.43, 95% CI: 2.98, 13.87, p=0.016, I2 =67.0%) more likely to be food insecure compared to households with small families.

**Conclusions:**

In this review, the prevalence of households and more than half of the households in Ethiopia had experienced food insecurity. Therefore, the food insecurity of households in Ethiopia continues to be a public health problem. The subgroup analysis showed that the Amhara region had the highest prone region for food insecurity and the size of the family was the potential significant factor for food insecurity in the household. Strategies to reduce household food insecurity must be implemented by sharing the best food secure region with the food insecurity region, community education and improving contraceptive use to reduce the number of family sizes.

The study registered on** t**he PROSPERO registration ID number is CRD42024532143.

**Supplementary Information:**

The online version contains supplementary material available at 10.1186/s40795-025-01207-x.

## Background

Food insecurity is defined as the limited or uncertain availability of nutritionally adequate and safe foods or the inability to acquire them in socially acceptable ways. Household food insecurity has multifaceted implications for individuals, families, and communities [1].

Food insecurity remains a significant public health concern globally, particularly in low- and middle-income countries like Ethiopia. Ethiopia, with a population of over 115 million, faces persistent food insecurity due to a variety of factors, including poverty, climate variability, conflict, and limited access to resources and infrastructure [2]. The country has experienced recurrent droughts and food crises, exacerbating vulnerability between rural and urban populations alike. In rural areas, where most Ethiopians live and depend on rainfed agriculture, fluctuations in agricultural productivity significantly affect food availability and access. Meanwhile, rapid urbanization has led to increased demand for food, often outpacing supply and affecting urban households’ ability to secure nutritious meals [3, 4].

Household food insecurity in Ethiopia is a complex phenomenon influenced by various individual, household, and contextual factors. Socioeconomic status, educational attainment, household composition, and access to social safety nets are among the key determinants shaping food insecurity status [5]. Gender dynamics also play a critical role, and women and children often bear the brunt of food insecurity due to unequal access to resources and decision-making power within households [6].

The consequences of household food insecurity in Ethiopia are far-reaching, affecting not only physical health but also mental well-being, educational attainment, and economic productivity. Malnutrition, particularly among women and children, is a pressing problem, contributing to high rates of stunting, wasting and micronutrient deficiencies [6]. Food insecurity also perpetuates cycles of poverty and undermines sustainable development efforts, hindering progress toward achieving national and international development goals, including Sustainable Development Goals (SDGs) [7].

Given the importance of addressing household food insecurity in Ethiopia, there is a growing body of research examining its prevalence, determinants, and consequences [8]. However, existing evidence is spread in various studies, with variability in magnitude and findings. For example, the results were inconsistent with a reported magnitude ranging from 20·5% o 93% [9] This variability underscores the need for a comprehensive synthesis of existing evidence to better understand the magnitude and drivers of food insecurity in households in the country. Therefore, this systematic review and meta-analysis aims to quantify the prevalence of household food insecurity in Ethiopia and identify its associated factors, offering a consolidated evidence base to inform policy making, targeted interventions, and future research.

## Methods and materials

### Study design

The systematic review and meta-analysis followed the guidelines outlined in the Statement of Preferred Reporting Items for Systematic Review and Meta-analysis Protocols (PRISMA-P). The PRISMA 2020 statement was used for reporting the findings [10]. The study protocol was registered in PROSPERO ID with the registration number CRD42024532143.

### Sources of information and search strategies

Sources of information were retrieved from different databases and search engines: such as PubMed, Scopus, Web of Science, African Journals online, EMBASE, Cochrane Library Google Scholar, and advanced Google from inception to November 2023 to identify relevant studies reporting on the level of household food insecurity in Ethiopia. The search strategy employed a combination of medical subject headings MeSH terms and key words were used to find the articles from listed databases by connecting Boolean operators: AND and OR. Consequently, ‘Household food insecurity’ OR ‘food insecurity’ OR ‘access’ AND ‘Prevalence’ AND ‘security’ OR ‘associated factors’ OR ‘risk factors’ OR ‘determinants’ OR ‘predictors’ AND ‘Ethiopia. We utilized medley software to download, rearrange, avoid duplications, review, and cite the studies. Additionally, manual searches of the reference lists of included studies and relevant review articles were conducted. The manual search was also conducted by authors (MB, EE, and MT) independently to check consistencies.

In our systematic reviews we used PEO (Population, Exposure, Outcome) frameworks as: -.

#### Population

Households in Ethiopia.

#### Outcome

Prevalence of food insecurity - is defined as the limited or uncertain availability of nutritionally adequate and safe foods or the inability to acquire them in socially acceptable ways [1].

#### Exposure/Factors

Socio-demographic or economic variables linked to food insecurity.

### Inclusion and exclusion criteria

The authors included original quantitative studies on the prevalence and factors associated with food insecurity in households. All published Ethiopian articles were searched from January1, 2017 to March 30, 2024, the general population, published in English on the topic of household food insecurity and its associated factors, while published articles without full text abstracts, editorials, letters, and articles that were not reported outcome variables or unrelated outcome variables for the interest of this study and a systematic review and meta-analysis study was not in the general household were excluded from the study.

### Data extraction and quality appraisal of the study

Data were extracted using the Joanna Briggs Institute (JBI) data extraction form and critical appraisal tools recommended for cross sectional studies [11]. Articles which were fulfilling the eligible criteria were entered into the Medley reference manager to identify duplicates and removal, as well as for a longer reference list. Data were extracted by a standard form developed in Microsoft Excel independently. For pooled prevalence of household food insecurity (first objective); author, year of publication, study region, study area, study design, sample technique, outcome measurement tool, sample size, and prevalence of the article. For associated factors (second objective): the variables and their data were extracted through two-by-two tables. Three independent authors (MB, EE and MT) extracted the data independently. During data extraction, differences between MB and EE authors were discussed to reach agreement and further supported by MT.

The quality assessment of the included studies was performed using the Joanna Briggs Institute (JBI) checklist [11], independently by two authors, with discrepancies resolved through discussion and consultation with other reviewers if necessary. The components of the nine parameters were: The sample frame appropriate for the target population (Q1), study participants sampled appropriately (Q2), adequate sample size (3), description of study participants and setting (Q4), data analysis with sufficient coverage of sample (Q5), are methods valid to identify conditions (Q6), reliable and standard condition measured for all participants (Q7), appropriate statistical analysis (Q8) and appropriate response rate management. This tool has nine questionnaires with yes, no unclear, and not applicable choices. One for yes responses and zero for unclear, not applicable, and no response was recorded. The sum score of yes for each questioner in a research was classified as low risk, which scored the quality index of seven and more were taken into account (Table 1).

(Table 1) - This table presents the quality assessment scores for each included study based on the JBI checklist criteria. Studies with a quality index score of seven or higher were classified as low-risk.

**Table 1:** Quality assessment results of eligible studies on household food insecurity and associated factors in Ethiopia, 2024: systematic and meta-analysis study

Author, Publication Year	Q1	Q2	Q3	Q4	Q5	Q6	Q7	Q8	Q9	Total score
Ayele AW, et al., 2020	Y	Y	Y	Y	Y	Y	Y	Y	Y	9
Shone M, et al., 2017	Y	Y	Y	Y	Y	N	Y	Y	Y	8
Tantu TA, et al., 2017	Y	Y	Y	Y	Y	Y	Y	Y	Y	9
Mulugeta M, et al., 2018	Y	Y	Y	Y	Y	Y	Y	Y	Y	9
Moroda GT, et al., 2018	Y	Y	Y	N	Y	Y	Y	Y	Y	8
Mota AA, et al., 2019	Y	Y	Y	Y	Y	Y	Y	Y	Y	9
Agidew AA, et al., 2018	Y	Y	Y	Y	Y	Y	Y	Y	Y	9
Sani S, et al., 2019	Y	N	Y	Y	Y	Y	Y	Y	Y	8
Samuel H, et al., 2019	Y	N	Y	Y	Y	Y	Y	Y	Y	8
Toma AA, et al., 2023	Y	Y	Y	Y	Y	Y	Y	Y	Y	9

*Key* *Y * Yes, *NR* Not reported, *NA* Not appropriate question codes

Q1. Was the sample frame appropriate to address the target population?

Q2. Were the study participants sampled in an appropriate way?

Q3. Was the sample size adequate?

Q4. Were the study subjects and the setting described in detail?

Q5. Was the data analysis conducted with sufficient coverage of the identified sample?

Q6. Were valid methods used to identify the condition?

Q7. Was the condition measured in a reliable and standard way for all participants?

Q8. Was there appropriate statistical analysis? and Q9. Was the response rate adequate and, if not, was the low response rate appropriately managed?

### Publication bias

Publication bias was determined by two main indicators; such as symmetrical graphical visualization studies within the triangle (funnel plot) and statically using the egger regression test with *p* > 0.05. If the two assumptions do not satisfy, there might be publications and further analysis needed to overcome such a problem.

#### Data analysis methods

The data extracted were imported into STATA 14 statistical software for the analysis of the prevalence of household food insecurity and associated factors in Ethiopia. A meta-analysis of the prevalence of food insecurity in households was carried out using a random effects model, which adjusts for observed variability, given its common application in meta-analyses (22).

To assess the potential risk of publication bias and the effects of small studies, funnel plots and Egger’s test (23, 24) were used. Heterogeneity between studies was examined using Cochrane Q-Static and I^2^ statistics.

Subgroup analyzes were performed to compare the pooled prevalence of household food insecurity and associated factors in different regions of Ethiopia. Pooled prevalence estimates were presented in forest plot format with the corresponding 95% confidence intervals. The categories level of I^2^ statistics are: 0% −40%, 30%−60%, 50%−90%, 75%−95% is mild, moderate, substantial, and considerable, respectively (5).

### Sensitivity analysis

 was performed to estimate the effect of each study on the overall estimate of the prevalence of household food insecurity and subgroup analysis.

## Certainty of evidence

The certainty of the evidence for the pooled outcomes was assessed using the GRADE approach, considering risk of bias, and publication bias.

## Results

### Identification, screening, and included studies

**Fig. 1:**
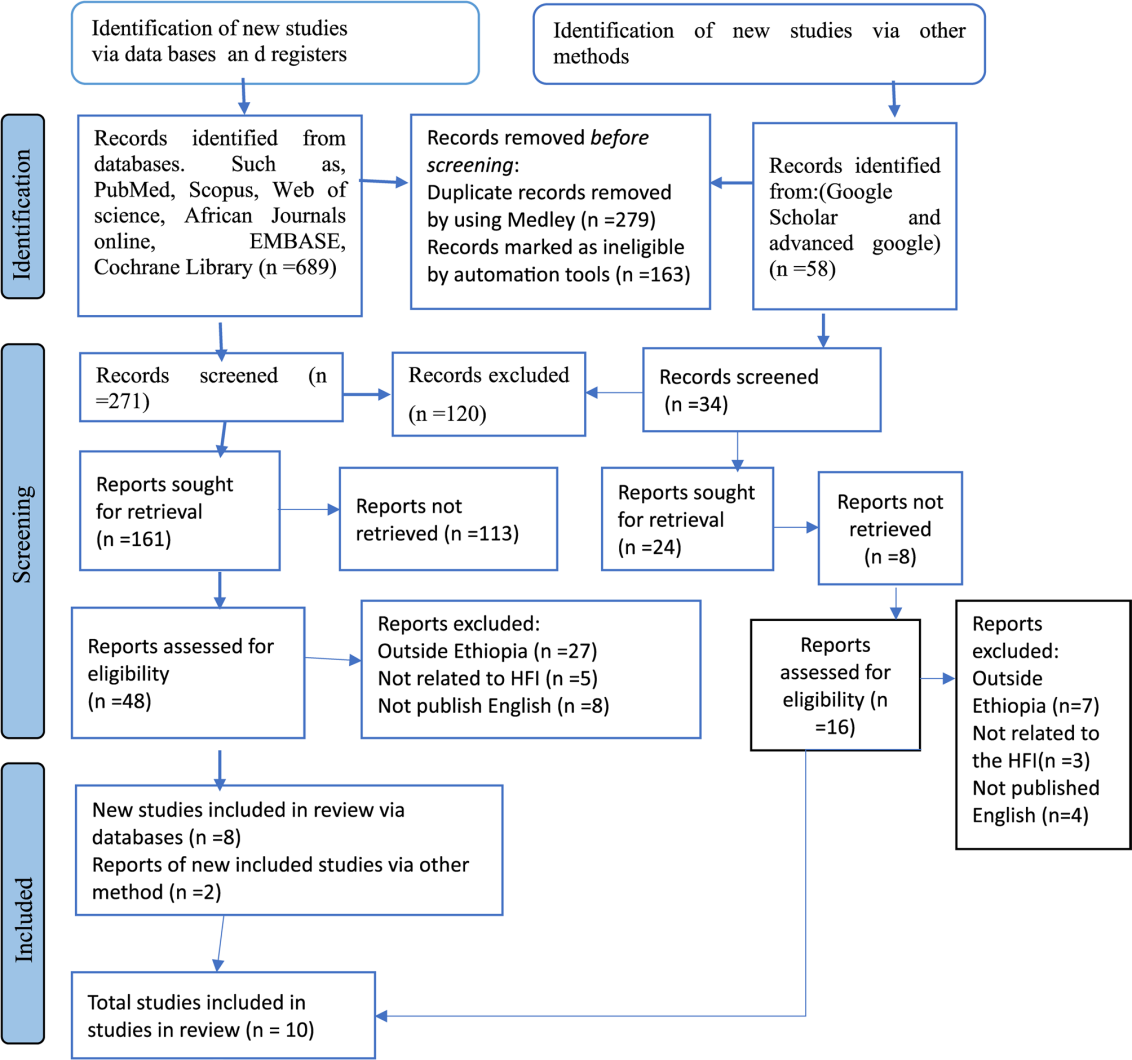
PRISMA flow diagram of the selection process on household food insecurity in Ethiopia, 2024

Our preliminary search was conducted based on the predetermined search strategies, which resulted in an overall 748 articles. Of this, we removed 279 duplicated articles and 163 records marked as ineligible by automation tools prior to further screening. In the next phase, we excluded 120 by title, abstract and irrelevant to the main topic: and publications repeatedly. Furthermore, 121 studies reports were not retrieved and the remaining 64 studies were checked for eligibility criteria to be included in this systematic review and meta-analysis. Only 10 articles were included for the final systematic review and meta-analysis because 54 articles were excluded for various reasons: Publication year, language of publication, study country, double articles, still main text not related to the topic (HFI) and in appropriate methodological approach (Fig. 1).

### Characteristics of the included studies in this systematic review and meta-analysis

We included ten articles that evaluated the pooled prevalence of food insecurity in households and its associated factors in Ethiopia [12–21]. These studies included a total of 4734 households. Two studies conducted in Amhara region [12, 18], four studies in SNNPR [13, 14, 17, 20], three studies in Oromia [15, 16, 21], and one study in Benshangul-Gumu [19]. All studies employed a cross-sectional study design. In our review, Four [13, 17, 18, 20], four [14, 15, 19, 21] and two [12, 16] articles, employed a simple random sampling, multistage sampling, and systematic techniques during data collection period, respectively. Regarding their data collection questionnaire, eight [13–18, 20, 21], one [19] and one [12] of the included articles used the HFIAS, CFSM and Food energy intake questionnaire, respectively. Response rates were reported by all studies (Table 2).

**Table 2:** Characteristics of the studies included in the meta-analysis of household food insecurity and its associated factors in Ethiopia, 2024

Author [Reference]	Year	Study Region	Study area	Study design	Sample Technique	Outcome measure tool	Sample size	Prevalence
Ayele AW, et al. [12]	2020	Amhara	East Gojjam districts	CS	SmRST	CFSM	504	43.25%
Shone M, et al. [13]	2017	SNNPR	West Abaya District	CS	SRS	HFIAS	779	38.10%
Tantu TA, et al. [14]	2017	SNNPR	Wolaita sodo town	CS	MSRS	HFIAS	609	37.60%
Mulugeta M, et al. [15]	2018	Oromia	Fedis district	CS	MSRS	HFIAS	773	58%
Moroda GT, et al. [16]	2018	Oromia	Boset district	CS	SmRST	HFIAS	397	21.70%
Motta AA, et al. [17]	2019	SNNPR	Damot Gale district	CS	SRS	HFIAS	155	71.60%
Agidew AA, et al. [18]	2018	Amhara	kutabere and Ambasel distrcts	CS	SRS	HFIAS	215	79.10%
Sani S, et al. [19]	2019	B.Gumuz	7 districts in Assosa zone	CS	MSRS	Food energy intake	276	53.62%
Samuel H, et al. [20]	2019	SNNPR	Areka town	CS	SRS	HFIAS	309	69.60%
Toma AA, et al. [21]	2023	Oromia	Arsi District	CS	MSRS	HFIAS	717	44.8%

*CFSM HFIAS *Household Food Insecurity* FEI: *Food Enegry Intake,* CS *Cross-Sectional* SmRST *Systematic Random Sampling Techniques* SRS *Simple Random Sampling* and MSRS *Multistage random sampling

The prevalence of household food insecurity was expressed as proportions (%) and pooled using random-effects meta-analysis, while associated factors were reported as odds ratios (ORs) with 95% confidence intervals.

### The pooled prevalence of household food insecurity in Ethiopia

**Fig. 2:**
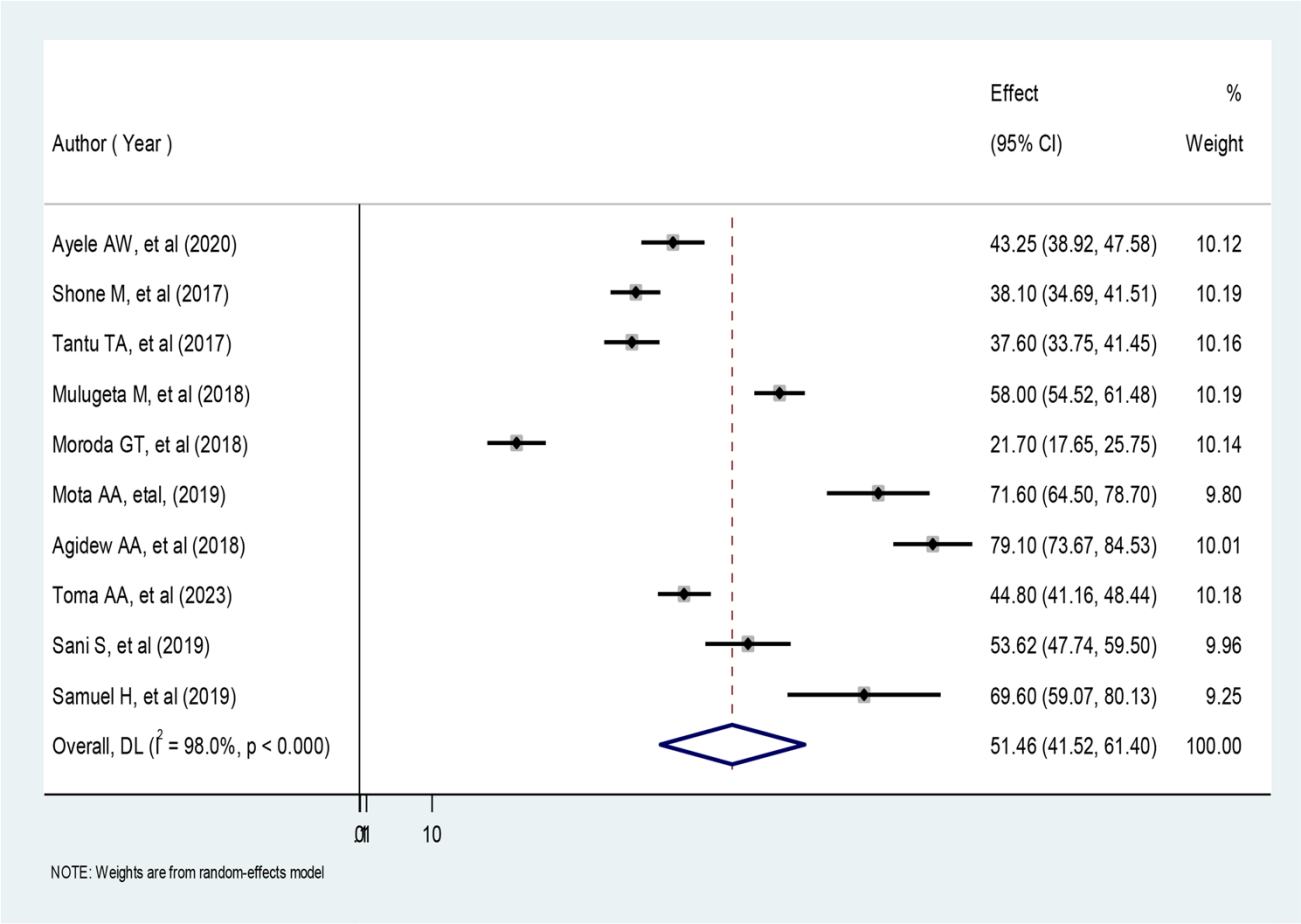
Forest plot of the pooled prevalence of household food insecurity in Ethiopia,2024

In our review, ten investigations concluded that more than half of households were food insecure in Ethiopia. According to this meta-analysis, the pooled prevalence of household food insecurity was 51.46% (95% CI: 41.52%, 61.40%). According to a random effects model with 95% CI, there was statistically significant heterogeneity among studies (I^2^ = 98.0%, *P* = 0.000) (Fig. 2). Therefore, these results showed that subgroup analysis was necessary due to significant heterogeneity among the primary studies [22].

**Fig. 3:**
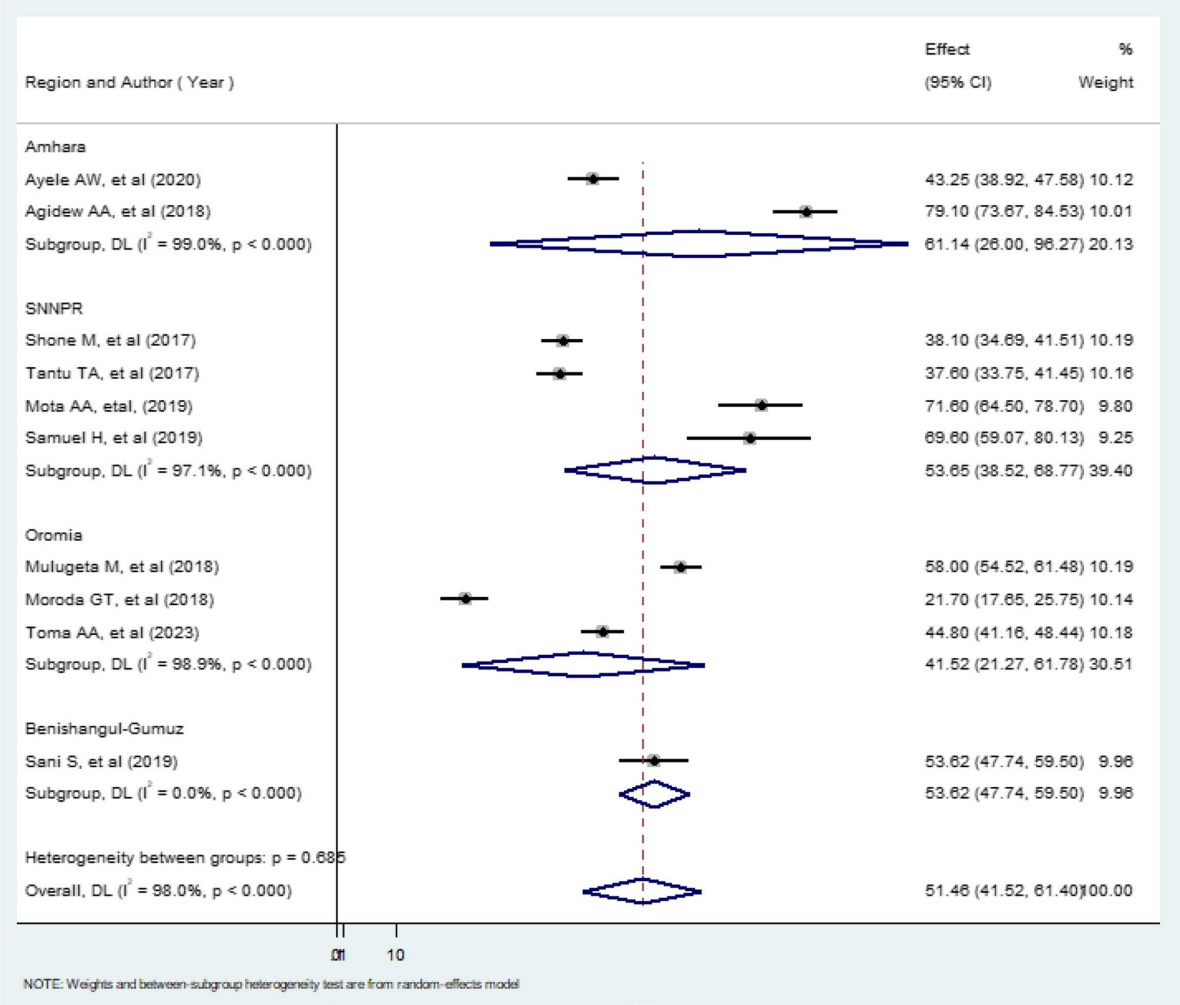
Subgroup analysis by regions on the pooled level of food insecurity at the household level

According to subgroup analysis by region, the highest prevalence of household food insecurity was observed in the Amhara region (61.14% (95% CI: 26.00, 68.77), I^2^ = 98.9%) while, the lowest prevalence of household food insecurity was observed in the Oromia region (41.52% (95% CI: 21.27, 61.78),I^2^ = 98.9%)(Fig. 3).

### Factors associating with household food insecurity

This study examined several types of factors associated with household food insecurity in Ethiopia. A total of ten studies were used to assess the prevalence of food insecurity in households and its associated factors in Ethiopia. As a result, some of the identified factors were investigated to assess the relationship between the household. Factors such as; age [15, 17], sex(two research’s([13, 17], education([12, 15], marital status in two researches [13, 14],, wealth index [17, 20], small land size in three articles [13, 15, 17], and not using agricultural inputs [17] were not significantly associated with food insecurity: However, the family size of the household was investigating from five articles [12, 13, 15, 17, 21] and significantly association with food insecurity.

**Fig. 4:**
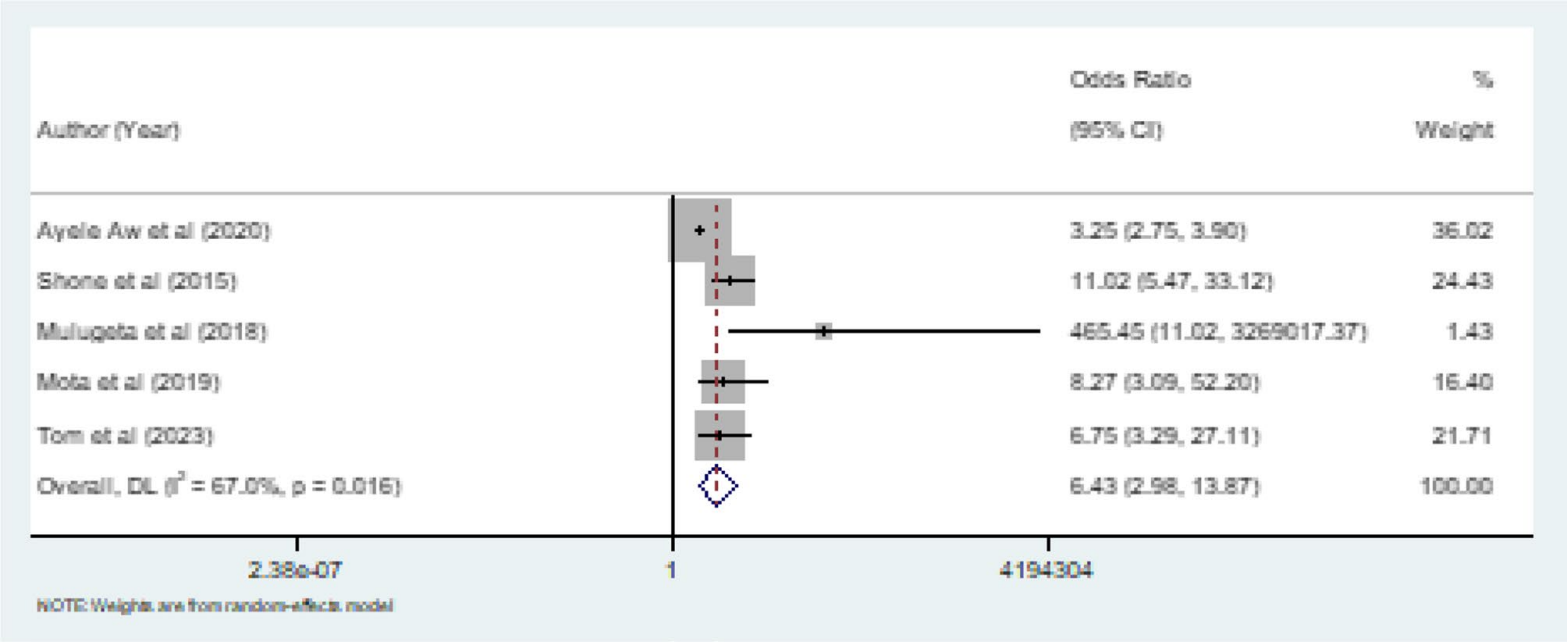
Pooled effect (OR) of the association between family size and household food insecurity in Ethiopia, 2024

Consequently, the result illustrated that households with a larger family size were 6.43 times (OR = 6.43, 95% CI: 2.98, 13.87, *p* = 0.016, I^2^ = 67.0%) more likely to be food insecure compared to households with a small family size (Fig. 4**).**

### Publication bias

**Fig. 5:**
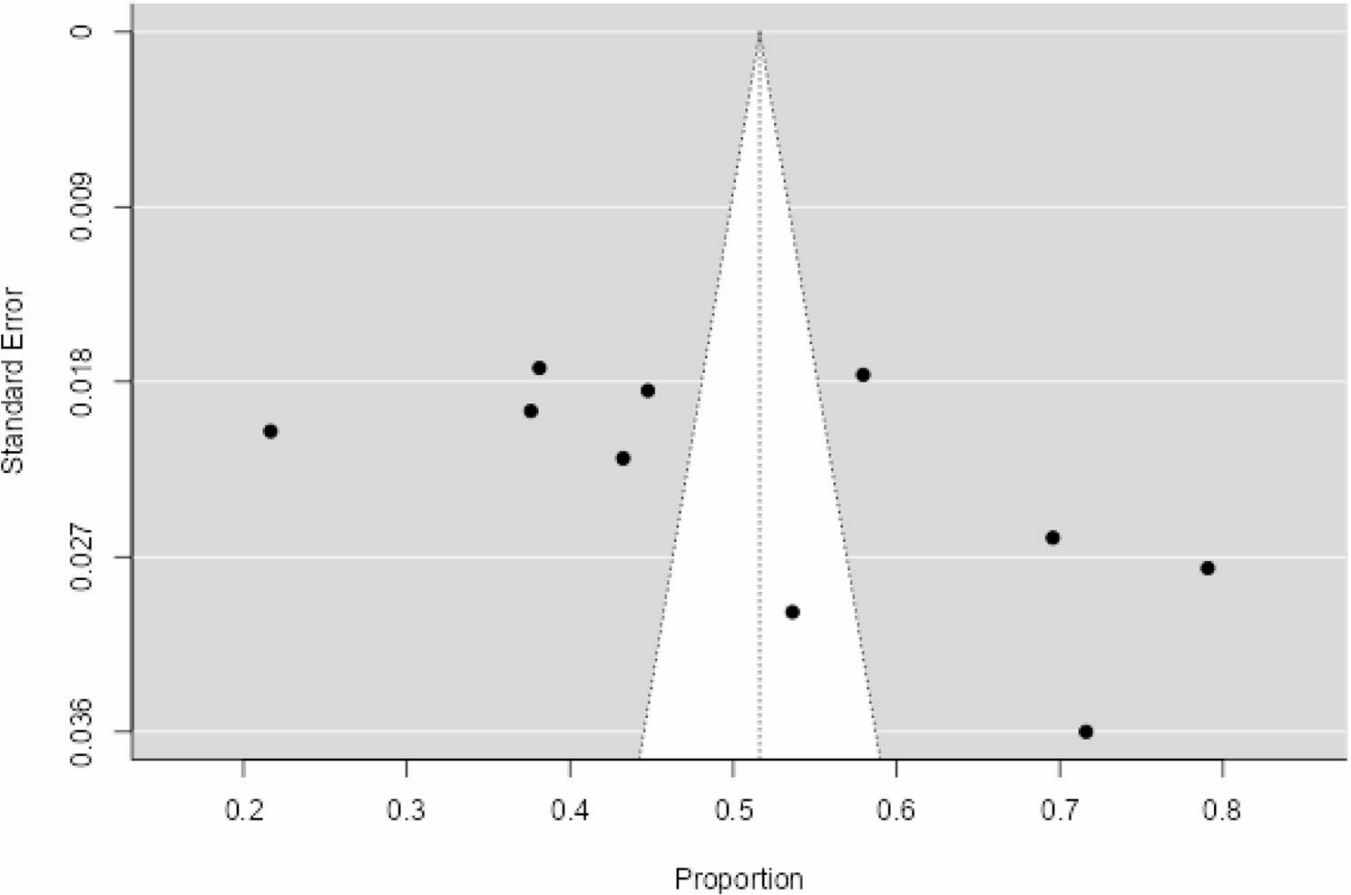
Funnel plot for publication bias of pooled prevalence of household food insecurity, 2024

We evaluated the funnel plot, which was used to visualize the presence or absence of publication bias. Thus, the visual examination of the funnel plot indicates that all of the studies were symmetrical distribution of included papers and suggests that distributed with lopsided (Fig. 5). At the same time, we checked the publication bias by using Egger’s test. The result of the eggers test was not statistically significant for the presence of publication bias (Kendall Tau = 0.378; *p* = 0.156). We can conclude that there was no publication bias in the included studies.

The result of this meta-analysis revealed statistically significant heterogeneity among studies (I^2^ = 98.0%; *p* = 0.000), we performed a subgroup analysis by region to adjust and reduce heterogeneity in addition to exploring potential sources of heterogeneity and examining whether the effect size varies between different subgroups (Fig. 3).

Moreover, we were conducting the factors that can cause sources of heterogeneity, and we conducted a meta-regression analysis, including sample size and publication year as covariates. However, in this review, the reports revealed that none of these variables had a significant impact on the observed heterogeneity between the studies (Table 3).

**Table 3:** Identify the factors that can affect heterogeneity among studies

Heterogeneity sources	coefficients	Standard errors	*p*-value
Sample size	3.451345	3.972534	0.854
Publication year	−1.063456	1.025438	0.0967

### Leave-One-Out sensitivity analysis

**Fig. 6:**
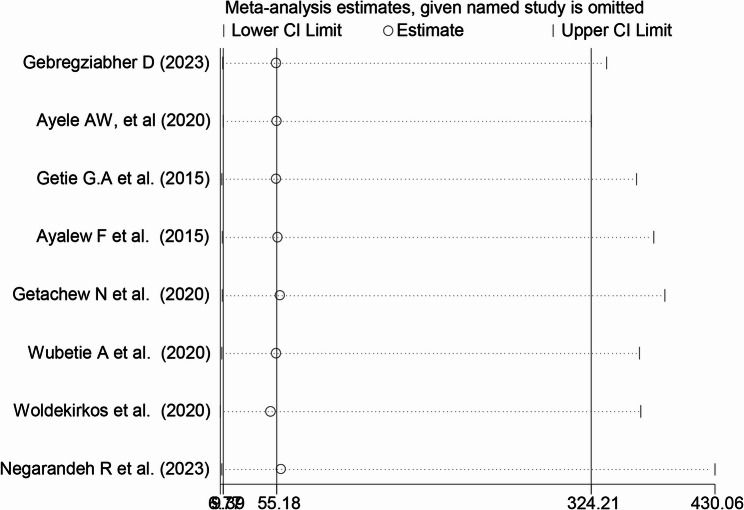
Leave out the sensitivity analysis for the study of household food insecurity and its associated factors in Ethiopia: A systematic review and meta-analysis, 2025

Leave-one-out sensitivity analysis was performed to assess the impact of individual studies on the overall pooled estimate of household food insecurity and its associated factors in Ethiopia. In this systematic review and meta-analysis, each study was sequentially excluded to assess its influence on the combined prevalence and related factors. The results demonstrated that omitting any single study did not cause a significant change in the total pooled estimates, indicating the robustness and stability of the findings. These results are visually depicted in Fig. 6, confirming that the general conclusions remain consistent despite the exclusion of individual studies.

## Discussion

The purpose of this systematic review and meta-analysis was to determine the pooled prevalence of household food insecurity in the country and its associated factors in Ethiopia. This systematic review and meta-analysis revealed that Ethiopian households experienced household food insecurity was 51.46% (95% CI: 41.52%, 61.40%) in a random effects model with 95% CI, statistically significant heterogeneity among studies (I^2^ = 98.0%, *P* = 0.000). This result was comparable to previous research conducted in East Africa (52%) [23], in southeastern Nigeria (53.5%) [24], Arak Iran (50.4%) [25] and 50.05% in national study of rural Ethiopia [26]. However, in this review of household food insecurity was lower compared to studies found in rural Iran 80% [27], in Northern India (77.2%) [28], Adana, Turkey (69%) [29]. This discrepancy might be due to comparative studies such as Iran’s repeated work and other studies; there might be a difference in socioeconomic status and social development.

The insecurity of household food in this review was greater in the studies conducted in USA (16%) [30], in Portuguese population 16.5% [31] and in Canada (13.8%) [32]. Thedifference might be due to the high economic stratus and variations in the methodological approach and measurement tool.

The meta-analysis revealed a statistically significant heterogeneity among the included studies (I² = 98.0%; *p* = 0.000), indicating substantial variability in the prevalence estimates of household food insecurity in Ethiopia. This heterogeneity could be due to differences in study settings, populations, measurement tools, or data collection periods. To explore potential sources of this variability, we performed a subgroup analysis by region. According to this analysis, Amhara had the highest prevalence of household food insecurity at 61.14% (95% CI: 26.00%, 68.77%), while Oromia reported a lower prevalence of 41.52%. The higher prevalence in Amhara can be attributed to the recurring events of drought, famine, and civil war that have affected much of the region [12, 18].

In this review, we found that the family size of the household was significantly associated with food insecurity in the household. Households that had a larger family size were 6.43 times more likely to be food insecure compared to households that had a small family size. This finding is consistent with studies reported in Osun State, Nigeria [33], and Ghana [34]. Other study from Esfahan, Iran, showed that food insecurity was positively associated with the number of members in the household [35]. When family size increases the share of household food also increases, leading to the scarcity of household food.

The high prevalence of household food insecurity found in this review reveals a critical public health and policy concern in Ethiopia. This underscores the need for integrated, multi-sectoral interventions targeting food security, including strengthening social protection programs, improving agricultural productivity, and addressing region-specific vulnerabilities such as recurrent drought in areas. The significant association between large family size and food insecurity suggests that family planning initiatives, combined with economic strengthening strategies at the household level, could play a vital role in reducing food insecurity. Furthermore, the marked disparity between Ethiopia and high-income countries points to the importance of addressing broader socioeconomic determinants and practicing measurement tools and policies in the local context for more effective responses.

### Conclusion and recommendation

Based on the results of this systematic review and meta-analysis, which revealed that more than half of the households in Ethiopia experienced food insecurity. Furthermore, based on the subgroup analysis, six in ten households experienced food insecurity in the Amhara region. Based on the results of this systematic review and meta-analysis, which revealed that more than half of households in Ethiopia experience food insecurity, with six in ten households affected in the Amhara region. We recommend the following targeted actions for households, government agencies, and stakeholders.

### Immediate (Short-term) interventions

Expand and strengthen the Productive Safety Net Program (PSNP) to cover more vulnerable households, especially in food-insecure regions such as Amhara. Ensure the timely and adequate distribution of food or cash transfers.

Emergency food aid and nutritional support during drought, conflict, or other periods of crisis, with a priority on high-risk areas.

Community-based nutrition programs focusing on children under five years of age, pregnant women, and lactating mothers.

### Sustainable (long-term) interventions

Promote and support the adoption of improved agricultural technologies (e.g. drought-resistant seeds, irrigation systems, climate-smart farming techniques).

Enhance access to affordable agricultural inputs, such as fertilizers and tools, through subsidies or credit schemes.

Invest in rural infrastructure, such as roads, markets, and storage facilities, to reduce post-harvest loss and improve market access.

Strengthen primary healthcare services to address malnutrition and food-related diseases, integrating them with agricultural extension programs.

### Policy and program development

Develop regional action plans that address the specific needs of areas with the highest food insecurity, such as Amhara, focusing on urban and rural contexts.

Design family planning and reproductive health programs that consider the link between household size and food insecurity, helping families better manage resources.

Integrate food security objectives into climate change adaptation strategies, given the significant role of environmental factors.

### More research needs

Conduct in-depth studies on the impact of family size and regional disparities on household food insecurity, including qualitative research to capture community perspectives.

Evaluate the effectiveness of current food security interventions to identify best practices and areas that need adjustment.

Explore the interplay between food insecurity and other social determinants (e.g. education level, gender dynamics) to inform multisectoral approaches.

## Supplementary Information

Supplementary material 1.

## Data Availability

The data sets generated and analyzed during the current study are available from the corresponding author on a reasonable request.

## Abbreviations

HFI: Household food insecurity
JBI: Joanna briggs institute
MeSH: Medical subject headings
PRISMA-P: Preferred reporting items for systematic review and meta-analysis protocols
